# Factors Influencing Vaccine Hesitancy in China: A Qualitative Study

**DOI:** 10.3390/vaccines9111291

**Published:** 2021-11-07

**Authors:** Jianli Wang, Qianqian Ji, Shuheng Dong, Shuangyu Zhao, Xinchen Li, Qiuqi Zhu, Sigui Long, Jingjing Zhang, Hui Jin

**Affiliations:** 1 Department of Epidemiology and Health Statistics, School of Public Health, Southeast University, Nanjing 210009, China; 213171070@seu.edu.cn (J.W.); 213170986@seu.edu.cn (Q.J.); 213172733@seu.edu.cn (S.D.); 213181074@seu.edu.cn (S.Z.); 213181160@seu.edu.cn (X.L.); 213181178@seu.edu.cn (Q.Z.); 213180365@seu.edu.cn (S.L.); 2 Key Laboratory of Environmental Medicine Engineering, School of Public Health, Southeast University, Ministry of Education, Nanjing 210009, China; 3 Department of Sociology, School of Humanities, Southeast University, Nanjing 210009, China; jingjingzhang@seu.edu.cn

**Keywords:** COVID-19, qualitative study, Chinese adults, vaccine hesitancy

## Abstract

Vaccine hesitancy has become a significant issue. We aimed to elucidate the factors influencing vaccine hesitation in Chinese residents and to analyze and recommend promotional strategies and measures. In total, 92 Chinese residents from 10 provinces were interviewed using semi-structured face-to-face interviews following a predetermined survey framework in this qualitative study. We found trust in vaccine safety, access to professional advice, and vaccine price and effectiveness to be the main factors influencing vaccine hesitation. Additionally, residents in areas with a higher per capita GDP tend to receive more social support, believe that vaccination is beneficial and can prevent diseases, pay more attention to whether the vaccine is safe and has undergone various clinical trials, and are more likely to seek advice from individuals with vaccination experience as opposed to their counterparts in areas with a lower per capita GDP. Notably, as per capita GDP rises, individuals become more concerned about the price of vaccines. Measures such as clarifying vaccine safety and effectiveness, reducing self-funded vaccine prices, offering free vaccination for special groups, strengthening the publicity role of medical staff, and taking advantage of network platforms are essential to reduce vaccine hesitancy among Chinese residents.

## 1. Introduction

As of 16 September 2021, there have been more than 22.6 million confirmed cases of coronavirus disease (COVID-19) globally, including 4.6 million deaths [1]. The COVID-19 pandemic poses tremendous challenges to human health, and the long-term effects of transmission-competent severe acute respiratory syndrome coronavirus 2 (SARS-CoV-2), which affects a variety of industries, are attracting great attention worldwide.

Based on the successful stories of smallpox eradication achieved by means of the vaccinia vaccine [2], heavy expectations have been placed on vaccines to suppress the COVID-19 pandemic. Unfortunately, although COVID-19 vaccines have been developed [3], its application in some countries is not promising [4,5]. Meanwhile, vaccine-preventable diseases have been resurging owing to the ever-growing anti-vaccination movement [6]. The promotion of vaccination is a thoughtful concern.

Vaccine hesitancy, according to the SAGE Working Group on Vaccine Hesitancy, is defined as a delay in acceptance or refusal of vaccination despite the availability of vaccination services [7]. Vaccine hesitancy hinders the realization of herd immunity and may prevent the function of vaccines in defending against diseases.

Previous late-stage trials have verified that CoronaVac (Sinovac, China), an inactivated and aluminum adjuvant vaccine, is 51% efficacious at counteracting COVID-19. Although WHO approved CoronaVac for emergency use on 1 June 2021, CoronaVac was authorized for conditional mass use to suppress the pandemic in China in early 2021 and is driving a domestic vaccination campaign [8]. The phase 1/2 clinical trials of three age groups (3–17, 18–59, and 60 and above) in China between 16 April and 2 December 2020 demonstrated that most adverse reactions were mild or moderate in severity and participants recovered within 2 days. The most common event was injection site pain [9,10,11]. CoronaVac phase 3 trial was carried out in Turkey between 14 September 2020 and 5 January 2021, with a vaccine efficacy of 83.5%. The most common systemic and local adverse event was fatigue and inoculation site pain, respectively. No deaths or grade 4 adverse reactions were observed during monitoring time [12]. The above clinical trial results supported that CoronaVac was effective and safe for defending against COVID-19.

In a subsequent online post-vaccination survey from February to March 2021 across China, approximately three-fifths reported no adverse events, whereas fatigue (19.2%) and local pain (18.5%) were the most reported reactions [13]. Another online survey on medical staff also showed a low incidence of adverse reactions, around 15%. The most common symptom was inoculation site pain (nearly 10%) [14]. Despite the efficacy and safety of the CoronaVac vaccine, Chinese residents still hold a different degree of vaccine hesitancy, which hinders the immunization campaign. Studies on vaccine hesitancy are required to explore the factors underlying vaccine delay or refusal.

Quantitative analysis (e.g., the 3C Model [15] and Structural Equation Model [16]) was applied to reveal factors that affect vaccine hesitancy. In contrast, the qualitative analysis of vaccine hesitancy remains to be strengthened. Qualitative analysis comprises in-depth investigation of the causes of phenomena using multiple data collection methods (e.g., interviews and observations), and conclusions are subsequently drawn from the obtained raw data. This study adopted a qualitative approach, which was guided by phenomenological methods and based on the principle of purposeful interviews. The aim of this study was to explore the factors influencing vaccine hesitancy among Chinese residents, the information serving to build an online vaccine health education platform to improve public vaccine confidence and to provide specific suggestions guiding the establishment of epidemic and disease prevention and control measures.

## 2. Materials and Methods

### 2.1. Study Design

Our study was conducted from 1 February to 31 March in 2021, when domestic cases were sporadic and imported cases were mostly under control in China. Additionally, the authorities introduced a series of policies to deal with the new stage of normalization. This non-experimental descriptive study used a designed vaccine survey framework to conduct interviews. To prevent researchers from interfering with the respondents’ views, open-ended questions were mainly used. The interviewer communicated with the interviewees according to the content of the survey framework.

The survey framework comprised two sections: (1) general information—mainly including the respondents’ age, gender, occupation, annual family income, and education level—and (2) vaccine hesitancy, based on the theory of planned behavior and the health belief model, and included three components, comprising a total of 12 open-ended questions: (1) participants’ awareness and attitudes toward the non-Expanded Program on Immunization (non-EPI), (2) other people’s influence, and (3) other factors affecting vaccination uptake. The framework of the factors influencing vaccine hesitancy in China that were mentioned by the interviewees is listed in Table 1; multiple categories and subcategories were subsequently created for each factor.

### 2.2. Data Collection

Semi-structured face-to-face interviews were conducted with the participants to analyze Chinese residents’ hesitation regarding the vaccine and its influencing factors. The interviewees were contacted prior to the interview to determine the interview time and place and to obtain consent for the recording of the interview. During the interviews, the interviewer remained neutral at all times.

### 2.3. Sampling Strategy

In total, 92 Chinese residents from 10 provinces (Jiangsu, Zhejiang, Shandong, Guangxi, Henan, Qinghai, Ningxia, Gansu, Xinjiang, and Hebei) were interviewed one-to-one. These interviewees were grouped into 4 groups of vaccinators at community health service centers, parents of children aged 0–6 years, adults aged 18–59 years, and adults 60 years and over. The general information of the interviewees is presented in Table 2.

### 2.4. Data Analyses

The recorded interview files were converted to text within 24 h following the end of the interview and subsequently analyzed using Colaizzi’s 7-step analysis method [10,17], and qualitative analysis software NVivo 11.0 (QSR International, Melbourne, Australia) was used to explore the factors affecting the interviewees’ vaccine hesitancy, recommend suggestions, and feasible measures, taking into consideration common themes.

During the research and design stage, the opinions of mentors and disease control professionals on the research topic and study design were obtained by conducting pre-interviews. The survey framework was consequently modified based on the feedback, so that formal interviews could be conducted smoothly. During the data collection stage, we paid attention to the colloquial presentation of the problem and remained neutral to ensure that the interview results truly reflected the interviewees’ ideas. During the data entry stage, the interview data were cleaned and verified, the definition of the classification framework clearly defined, and the data coded to ensure a clear operational definition (coded twice, by two separate researchers, using the NVivo 11.0 software); the internal consistency was also verified. When coding opinions differed, the views were unified after discussion.

## 3. Results

A total of 92 participants, from 10 Chinese provinces with different gross domestic product (GDP) levels, attended the face-to-face interviews. Among them, there were 15 healthcare workers, 22 parents of children aged 0–6 years, 34 adults aged 18–59 years, and 21 adults aged 60 years and over. Table 2 presents the detailed sociodemographic information of the participants.

**Figure 1:**
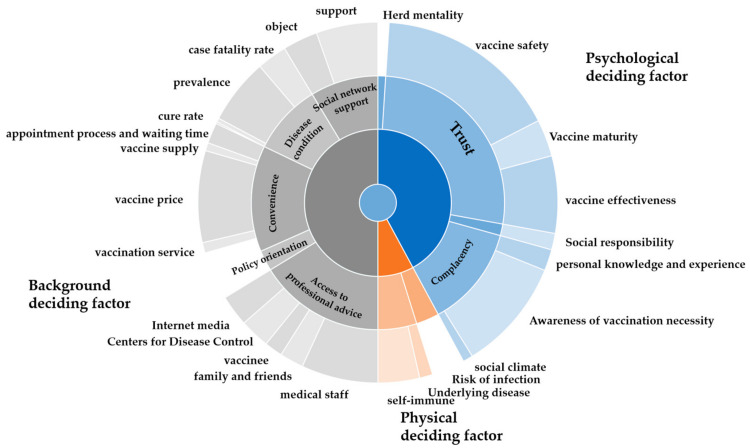
Wheel diagram of factors influencing vaccine hesitancy in China.

According to the content of the responses to the open-ended questions, the qualitative data were classified into three themes: (1) background determinant factors, (2) physical determinant factors, and (3) psychological determinant factors; all three themes had a series of sub-themes. These are listed in Table 1 and were further analyzed, as described below. The summary of factors influencing vaccine hesitancy in China was shown in Figure 1 and Supplementary Table S1.

### 3.1. Trust in Vaccine Safety

Trust in vaccine safety (among the psychological determinant factors) was the most influential factor contributing to vaccine hesitancy among the four groups of interviewees. The safety of vaccines was referred to 191 times (Table 3) when asked about the biggest worry regarding being vaccinated; terms such as “side effects”, “adverse reactions”, and “safety” were repeatedly mentioned. This factor was well-described by one participant, “First of all, the vaccine production process must be guaranteed to be safe. Recently, there have been many reports on fake vaccines and virulent vaccines. The public’s view of vaccines is actually not optimistic, and it may be blamed on vaccine safety”. The majority of participants were concerned about adverse reactions after vaccination; specific examples included allergies, hay fever, and blindness. In particular, the groups concerning adults aged 60 years and over and children aged 0–6 years, who may have lower physical fitness levels, along with physical health issues, tended to be highly focused on this theme. This was highlighted by one participant, “Take COVID-19 as an example. Through the Internet, I learned that vaccination may lead to adverse reactions, such as allergic reactions and even blindness; the consequences are very serious”. Meanwhile, some healthcare workers were worried about unsafe storage and maintenance of the cold chain, although they understood the experimental procedure of vaccine development and mentioned that “more information about the adverse reactions to vaccination is in huge demand to guide informed opinions”. On the one hand, previous negative reports regarding vaccine safety affected respondents’ trust in vaccine safety, while, on the other hand, people’s own psychological prejudice played an important role. That is, in addition to the risk of illness, people emphasize the fear of developing possible side effects after vaccination. Together, these factors have contributed to the growing crisis of mistrust in vaccines. One parent mentioned, “If vaccines do not really prevent diseases but do produce side effects, then the loss outweighs the gain”. The safety of vaccines is of great importance, and there was a consensus that there is a lack of safety and, therefore, a preference to avoid vaccination.

### 3.2. Access to Professional Advice

As recipients of information from multiple sources, the greatest source of advice reported by these four groups was healthcare workers compared with online media, family, and friends, and those who have already been vaccinated. Access to professional advice from healthcare workers played a critical role in individuals’ decision regarding whether to be vaccinated; it was mentioned 80 times in total (Table 3). Notably, this advice was recognized by the 92 participants as “professional”, “constructive”, “well-accepted,”, “decisive”, and “official”. Most interviewees were inclined to seek vaccination advice from professional healthcare workers. For example, one parent of children aged 0–6 years stated, “Their positive guidance has a decisive effect on us, and if a doctor recommends to have my kids vaccinated, I would definitely do it”. Some even expressed, “I just listen to what the doctors say as they are appointed by the country. I feel confident to trust them”. It is noteworthy that healthcare workers, whose behavior greatly impacts the behaviors and attitudes of the public, could benefit or bring disadvantage to vaccination rates through social norms due to their particular identities and actions as role models. For example, one older adult stressed, “Some healthcare workers may not take it themselves but they will recommend us to get vaccinated, so we may feel a little confused and hesitant”. Moreover, some adults expressed opinions such as, “We don’t know when vaccination is appropriate, and there are no professional workers devoted to clarifying the situation to us. The channels for us to consult are also very limited. We hope that the Centers for Disease Control and Prevention can publicize more information about vaccines”. Comprehensive interventions for healthcare workers, including education, training courses, and incentives after inoculation, are essential. The Internet was another information source that had a decisive effect on the decision whether to be vaccinated. One parent mentioned, “We will also vaccinate our children based on health literacy obtained from the official online media”.

### 3.3. Vaccine Price

The vaccine price was the second largest contributor to vaccine hesitancy, as determined by participant responses (apart from older adults aged 60 years and over). Among them, 19 (55.9%) adults aged 18–59 years and 14 (63.6%) parents of children aged 0–6 years expressed vaccine price as a factor affecting vaccination uptake. Although most interviewees were aware of the necessity of non-EPI vaccines, the vast majority stated that the price would significantly affect their decision in view of family income and personally acceptable expectations regarding medical expenditure. For example, one parent stressed, “If the vaccine price is too high, I may weigh vaccinating my child against affordability”. Respondents generally associated vaccine prices with the necessity for vaccination; that is, if vaccination is necessary, the price has nearly no effect, whereas, if not, the price has a great influence on their decision. One adult stated, “Though the human papillomavirus 9-valent vaccine was proven effective and safe, considering that college students’ income is relatively limited, we may hesitate being vaccinated as it is a big financial burden”. Most respondents expressed hope: “The price of non-EPI vaccines could be controlled at a level acceptable to the public, and the most desired measure is for more of the cost to be reimbursed by health insurance schemes”. However, in this study, vaccine price had little impact on older adults aged 60 years and over.

### 3.4. Vaccine Effectiveness

The third largest contributing factor to vaccine hesitancy in adults aged 18–59 years (18, 52.9%) and parents of children aged 0–6 years (12, 54.5%) was vaccine effectiveness. These participants had specific concerns regarding effectiveness, including the extent of protection against different emerging vaccine strains, effective vaccine cycles, specific vaccine responses in different populations, and ineffective vaccination due to virus mutation. For example, one participant stated, “Take the flu vaccine as an example. Given that a series of flu viruses exist, can a single shot of the vaccine cover most virus varieties? If it can, I will take it. If it can cover only one or two varieties, I think it makes little sense”. Another mentioned, “I’m worried that, even if I have been vaccinated, there is still a chance of infection”. Some respondents expressed hope that relevant agencies would publish more specific data on the effectiveness of vaccines to relieve their concern. Moreover, healthcare workers and older adults aged 60 years and over showed little concern regarding vaccine effectiveness.

### 3.5. Other Factors

The prevalence of the disease affected the willingness of healthcare workers, older adults (12, 57.1%), and parents of children aged 0–6 years (12, 54.5%) to consider vaccination. For example, the recent COVID-19 pandemic has led to a consensus in society, enabling individuals to evaluate the pros and cons and make reasonable judgments on vaccination demand based on the characteristics of the infectious disease. One older participant stated, “If the COVID-19 outbreak becomes more widespread, I will be more worried and panicky; the prevalence of COVID-19 should be the main consideration”. Another stressed, “Widespread prevalence and a high case fatality rate of influenza will push me to be vaccinated”. Self-immunity and policy directions were also frequently mentioned by older participants. Age-related immune dysfunction increases the prevalence of infection and autoimmune disease in the older population. In addition, they have great trust in the national policy orientation. For example, “I am not worried about vaccination at all, as long as it’s a national call”. Additionally, one party member stressed, “My age has led to it being a habit for me to do what is demanded by the Communist Party of China without any concerns”. Therefore, the relevant national policies regarding vaccination include coordinating various interventions in terms of health protection, social and economic needs, and safety and cost requirements and lead to the priority status given to vaccination being significant for adherence.

### 3.6. Regional Differences

**Figure 2:**
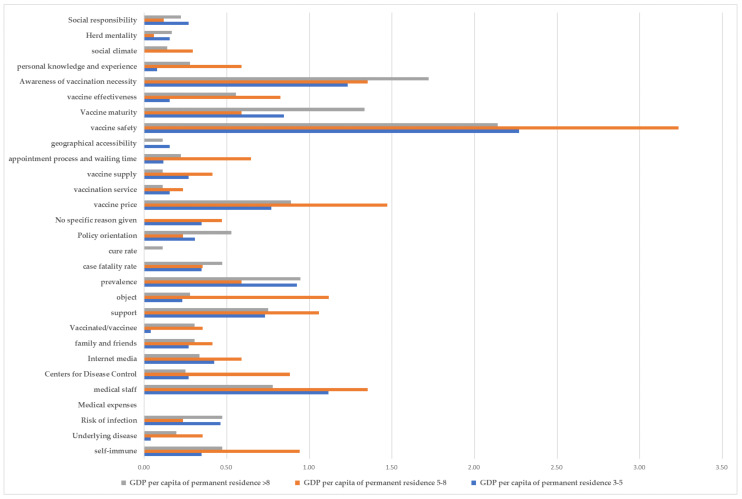
Frequency distribution of factors influencing vaccine hesitancy based on the 2020 per capita gross domestic product of each province according to the National Bureau of Statistics of China [18].

According to the per capita GDP obtained from the China Statistical Yearbook 2020, compiled by the National Bureau of Statistics [18], the included regions were divided into groups representing a GDP of (1) higher than 80,000 yuan (Jiangsu, Zhejiang, and Chongqing), (2) 50,000–80,000 yuan (Shandong, Henan, Ningxia, and Xinjiang), and (3) 30,000–50,000 yuan (Qinghai, Hebei, Guangxi, and Gansu), represented in Figure 2. It was found that residents in areas with a higher per capita GDP received more social support. Additionally, residents in these areas believed that vaccination is more beneficial to the body and can prevent diseases, they paid more attention to whether the vaccine is reliable and has undergone a large number of clinical trials, and were more likely to seek advice from individuals with vaccination experience. Notably, concurrent with an increase in per capita GDP, there was increasing concern regarding the vaccine price.

## 4. Discussion

This study explored the factors influencing vaccine hesitancy among Chinese residents in order to provide recommendations to guide the establishment of epidemic and disease prevention and control measures. We found the main factors influencing vaccine hesitancy in this population to be trust in vaccine safety, access to professional advice, and vaccine price and effectiveness. While vaccine hesitancy is a global phenomenon, the factors that affect it vary from country to country [19]. The situation in Europe is similar to that in China, where several studies have shown that serious adverse effects of vaccination, such as thrombosis and thrombocytopenia and other severe vascular adverse outcomes have caused controversy among the public and slowed down the European vaccine program [20,21,22,23]. Italy and France reported higher levels of hesitation, possibly because of a lack of confidence in the safety of vaccines due to historically negative coverage [24,25,26]. In the United States, concerns about side effects and safety vaccines, lack of trust in government, and concerns about rapid development are the main reasons for delaying vaccination [27]. Moreover, residents in areas with a higher per capita GDP tended to receive more social support and have more trust in the benefits and function of vaccines in disease prevention as opposed to their counterparts from areas with a lower per capita GDP.

Our study reveals that there are similarities and differences in the factors influencing vaccine hesitancy among the four groups of respondents, which can greatly benefit targeted vaccine promotion measures among different population groups. Apart from in adults aged 60 years and over, the four main factors affecting vaccine hesitation were vaccine safety, necessity, effectiveness, and price. The factors affecting vaccine hesitancy in the older population differed from those affecting the other groups. In addition to vaccine safety and necessity, older adults paid more attention to the prevalence of a disease and national policy guidance; therefore, when formulating relevant measures, we should take into account these differences to promote science popularization. All four groups were more likely to seek vaccination advice from professional health care and disease control personnel, although mainly from health care personnel.

Overall, psychological determinants were the main factors influencing vaccine hesitancy in this study. Based on the 3C Model of vaccine hesitancy, it is known that the safety and effectiveness of a vaccine affects the degree of trust, and the need for vaccination impacts complacency. Therefore, relevant measures should be taken when divulging scientific publicity against vaccine hesitancy. We found that residents in areas with a higher per capita GDP are more supported in terms of social networks and have a deeper recognition of the need for vaccination than those in areas with a lower per capita GDP. Vaccine reliability and vaccination experience are important factors that influence choices. However, with an increase in per capita GDP, people are increasingly concerned about the price of a vaccine.

According to the results of the conducted interviews, our recommendations to reduce vaccine hesitancy are as follows.

### 4.1. Ensure Vaccine Safety and Effectiveness

Vaccine manufacturers and related agencies should ensure the safety of vaccines, prevent the occurrence of safety incidents, and provide adverse reaction information to ensure public confidence in the vaccine [28]. Additionally, authorities concerned are recommended to investigate potential complications of vaccines and disclose the results to the public in a transparent manner [29]. Furthermore, vaccine regulators should strengthen safety management and continuous monitoring of vaccines and actively disclose evaluation data, such as vaccine quality, safety, and effectiveness [30].

### 4.2. Reduce the Price of Self-Funded Vaccines and Provide Free Vaccination to High Risk Groups

Along with rapid economic development, the delivery of more self-funded vaccines, such as free vaccines, should be considered. This can also be accomplished by health insurance schemes partially subsidizing self-funded vaccines to lower the financial burden of vaccination. Some large cities in China have implemented the policy of free influenza vaccination for high-risk groups, such as older adults and individuals with chronic diseases, which has effectively increased the influenza vaccination rate [31]. In addition, free vaccination for frontline medical staff is also worth considering. An investigation in the United States showed that, in the case of non-compulsory influenza vaccination of medical staff, whether the vaccine provided by institutions is free or not will greatly affect the willingness of medical staff to receive it [32].

### 4.3. Strengthen the Publicity Role of Medical Staff

It is suggested that the relevant knowledge of grassroots doctors should be strengthened through training in order to increase the public’s trust in vaccines [33]. Similarly, studies based on the current state of vaccine hesitancy in the United States show that a biggest booster of vaccine confidence is due to a doctor’s advice, and this can be reinforced by mainstream media rebuttal of misinformation [34]. Furthermore, vaccine publicity can be combined with existing chronic disease management plans and the community doctor service model; the corresponding prescribed suggestion list can be issued with scientific publicity to guide specific patients to take the initiative to get vaccinated [35].

According to the results of the interviews conducted in this study, social network platforms are also one of the channels where interviewees obtained vaccine-related information. Therefore, public health institutions or health care personnel could seize the opportunity to popularize scientific knowledge related to vaccines on these platforms, deal with negative responses to vaccines and safety issues, and expand their own influence to help increase the public’s trust in the safety and effectiveness of vaccines [36].

## 5. Conclusions

It is important to reflect on the limitations of this study. First, our study used purposive sampling; therefore, the respondents are not fully representative of the general population. More than 85% of the participants had a high school diploma or higher. Individuals with advanced educational diplomas may have more positive and favorable views than others. Second, our study was conducted from 1 February to 31 March in 2021, and new changes to the national policy and the epidemic situation have occurred after this, which may affect attitudes toward vaccine hesitancy. Third, no participants specifically declined to be interviewed; thus, we cannot rule out that these individuals were inherently more supportive and had more favorable views than others.

Through qualitative analysis, we identified the factors influencing vaccine hesitation among Chinese residents. Our findings can provide a basis for the construction of online vaccine health education platforms and the improvement of public confidence in vaccines. Moreover, our specific suggestions can guide the selection of prevention and control measures for epidemic diseases.

## Figures and Tables

**Table 1:** Overview of factors influencing vaccine hesitancy in China mentioned by interviewees.

Theme	Sub-Theme
Background deciding factor	Disease condition
	case fatality rate; prevalence; cure rate
	Social network support
	object/support
	Convenience
	vaccination service; vaccine supply; vaccine price
	geographical accessibility
	appointment process and waiting time
	Policy orientation
	Access to professional advice
	Centers for Disease Control; medical staff
	family and friends; Internet media
	vaccinee
Physical deciding factor	Risk of infection
	Physical conditions
	Underlying disease; self-immune
	Medical expenses
Psychological deciding factor	Herd mentality
	Social responsibility
	Trust
	vaccine safety; Vaccine maturity; vaccine effectiveness
	Complacency
	personal knowledge and experience
	Awareness of vaccination necessity
	social climate

**Table 2:** Participants’ sociodemographic information.

Demographic Characteristics	Healthcare Workers	Adults Aged 18–59	Older People Over 60	Parents of Children Aged 0–6
Gender	-	-	-	-
Male	4 (26.7)	13 (38.2)	11 (52.4)	6 (27.3)
Female	11 (73.3)	21 (61.8)	10 (47.6)	16 (72.7)
GDP per capita of permanent residence (RMB 10,000)	-	-	-	-
3–5	4 (26.7)	13 (38.2)	7 (33.3)	6 (27.3)
5–8	2 (13.3)	14 (41.2)	3 (14.3)	8 (36.4)
>8	9 (60.0)	7 (20.6)	11 (52.4)	8 (36.4)
Educational level	-	-	-	-
elementary school or below	0 (0.0)	0 (0.0)	3(14.3)	0 (0.0)
Junior high school	0 (0.0)	3 (8.8)	7(33.3)	0 (0.0)
High school graduate or equivalent	1 (6.7)	4 (11.8)	5 (23.8)	3 (72.7)
College or equivalent	13 (86.7)	24 (70.6)	6 (28.6)	16 (72.7)
Master’s Diploma or above	1 (6.7)	3 (8.8)	0 (0.0)	3 (72.7)
Annual household income (RMB 10,000)	-	-	-	-
<5	2 (13.3)	5 (14.7)	3 (14.3)	2 (9.1)
5–10	1 (6.7)	15 (44.1)	10 (47.6)	8 (36.4)
11–15	4 (26.7)	6 (17.6)	1 (4.8)	5 (22.7)
>16	8 (53.3)	8 (23.5)	7 (33.3)	7 (31.8)
Occupation	-	-	-	-
Government agencies and institutions		4 (11.8)	0 (0.0)	6 (27.3)
Business/enterprise		2 (5.9)	1 (4.8)	8 (36.4)
Production staff/worker		4 (11.8)	0 (0.0)	4 (18.2)
Full-time student		23 (67.6)	0 (0.0)	0 (0.0)
Retired		0 (0.0)	19 (90.5)	0 (0.0)
Else		1 (2.9)	1 (4.8)	3 (13.6)
None		0 (0.0)	0 (0.0)	1 (4.5)
Number of children				-
1				12 (54.5)
2				10 (45.5)
Has the child played in the last year influenza vaccine				-
Yes				10 (45.5)
No				12 (54.5)

**Table 3:** The most frequently mentioned factors influencing vaccine hesitancy in Chinese residents.

Themes	Sub-Themes	Frequency (%) ^1^
Background deciding factor	case fatality rate	32 (34.8)
	prevalence	68 (73.9)
	vaccine price	77 (83.7)
	appointment process and waiting time	22 (23.9)
	vaccine supply	18 (19.6)
Physical deciding factor	risk of infection	33 (35.9)
	self-immune	42 (45.7)
Psychological deciding factor	social responsibility	17 (18.5)
	vaccine safety	191 (>100)
	vaccine effectiveness	80 (87.0)
	awareness of vaccination necessity	117 (>100)

^1^ Total number of participants was 92. Participants gave more than one response, so totals do not equal 100.

## Supplementary Materials

The following are available online at https://www.mdpi.com/article/10.3390/vaccines9111291/s1, Supplementary Table S1. An overview of factors influencing vaccine hesitancy in China.
