# Supplementary material for: Factors Influencing Vaccine Hesitancy in China: A Qualitative Study
Source: Vaccines (Basel). 2021 Nov 7;9(11):1291. doi: 10.3390/vaccines9111291 (PMC8622488; doi:10.3390/vaccines9111291)
Supplement: Supplementary file 1 [file vaccines-09-01291-s001.zip › vaccines-1431655 supplementary.pdf]

Supplementary Table S1. A overview of factors influencing vaccine hesitancy in China.

			Parents of children aged 0-6	Older people over 60	Adults aged 18-59	Healthcare workers
**Physical deciding factor**	**Physical conditions**	**self-immune**	17(40.5)	13(31)	10(23.8)	2(4.8)
		**Underlying disease**	1(7.1)	7(50)	4(28.6)	2(14.3)
	**Risk of infection**		9(27.3)	8(24.2)	8(24.2)	8(24.2)
	**Medical expenses**		0(0)	0(0)	0(0)	0(0)
**Background deciding factor**	**Access to professional advice**	**medical staff**	29(36.3)	15(18.8)	25(31.3)	11(13.8)
		**Centers for Disease Control**	12(36.4)	5(15.2)	10(30.3)	6(18.2)
		**Internet media**	13(39.4)	4(12.1)	13(39.4)	3(9.1)
		**family and friends**	6(30)	0(0)	11(55)	3(15)
		**vaccinee**	13(61.9)	3(14.3)	5(23.8)	0(0)
	**Social network support**	**support**	22(34.4)	8(12.5)	25(39.1)	9(14.1)
		**object**	17(48.6)	1(2.9)	13(37.1)	4(11.4)
	**Disease condition**	**prevalence**	14(20.6)	15(22.1)	24(35.3)	15(22.1)
		**case fatality rate**	11(34.4)	3(9.4)	10(31.3)	8(25)
		**cure rate**	1(25)	0(0)	3(75)	0(0)
	**Policy orientation**		8(25.8)	14(45.2)	4(12.9)	5(16.1)
	**Convenience**	**No specific reason given**	7(36.8)	1(5.3)	9(47.4)	2(10.5)
		**vaccine price**	20(26)	7(9.1)	35(45.5)	15(19.5)
		**vaccination service**	1(8.3)	3(25)	5(41.7)	3(25)
		**vaccine supply**	6(33.3)	2(11.1)	4(22.2)	6(33.3)
		**appointment process and waiting time**	4(19)	2(9.5)	11(52.4)	4(19)
		**geographical accessibility**	5(55.6)	3(33.3)	1(11.1)	0(0)
**Psychological deciding factor**	**Trust**	**vaccine safety**	66(34.6)	30(15.7)	58(30.4)	37(19.4)
		**Vaccine maturity**	25(31.3)	7(8.8)	31(38.8)	17(21.3)
		**vaccine effectiveness**	15(39.5)	4(10.5)	9(23.7)	10(26.3)
	**Complacency**	**Awareness of vaccination necessity**	40(34.2)	26(22.2)	32(27.4)	19(16.2)
		**personal knowledge and experience**	11(50)	2(9.1)	3(13.6)	6(27.3)
		**social climate**	2(20)	0(0)	5(50)	3(30)
	**Herd mentality**		4(36.4)	3(27.3)	4(36.4)	0(0)
	**Social responsibility**		0(0)	9(52.9)	7(41.2)	1(5.9)
